# The distribution pattern of 5-methylcytosine in rye (*Secale* L.) chromosomes

**DOI:** 10.1371/journal.pone.0240869

**Published:** 2020-10-15

**Authors:** Anna Kalinka, Magdalena Achrem

**Affiliations:** 1 Institute of Biology, University of Szczecin, Szczecin, Poland; 2 Molecular Biology and Biotechnology Center, University of Szczecin, Szczecin, Poland; Leibniz-Institute of Plant Genetics and Crop Plant Research (IPK), GERMANY

## Abstract

The rye (*Secale* L.) genome is large, and it contains many classes of repetitive sequences. *Secale* species differ in terms of genome size, heterochromatin content, and global methylation level; however, the organization of individual types of sequences in chromosomes is relatively similar. The content of the abundant subtelomeric heterochromatin fraction in rye do not correlate with the global level of cytosine methylation, hence immunofluorescence detection of 5-methylcytosine (5-mC) distribution in metaphase chromosomes was performed. The distribution patterns of 5-methylcytosine in the chromosomes of *Secale* species/subspecies were generally similar. 5-methylcytosine signals were dispersed along the entire length of the chromosome arms of all chromosomes, indicating high levels of methylation, especially at retrotransposon sequences. 5-mC signals were absent in the centromeric and telomeric regions, as well as in subtelomeric blocks of constitutive heterochromatin, in each of the taxa studied. Pericentromeric domains were methylated, however, there was a certain level of polymorphism in these areas, as was the case with the nucleolus organizer region. Sequence methylation within the region of the heterochromatin intercalary bands were also demonstrated to be heterogenous. Unexpectedly, there was a lack of methylation in rye subtelomeres, indicating that heterochromatin is a very diverse fraction of chromatin, and its epigenetic regulation or potential influence on adjacent regions can be more complex than has conventionally been thought. Like telomeres and centromeres, subtelomeric heterochromatin can has a specific role, and the absence of 5-mC is required to maintain the heterochromatin state.

## Introduction

DNA methylation is a crucial epigenetic mechanism that plays a role in the regulation of the eukaryotic genome. It is widely believed that DNA methylation appeared during evolution as a protective mechanism against the proliferation and translocation of transposable elements (TEs) in eukaryotic cells [1]. In rye, the TE content is estimated to be ~70%, with retrotransposons making up the majority of this fraction [2]. Their hypermethylation contributes significantly to the global level of DNA methylation. In the *Arabidopsis* genome, the entire sequences of TEs are methylated in all sequence contexts, i.e. CG, CHG (H = A, C or T), and CHH [3]. The epigenetic silencing state of TEs ensures genomic stability. However, stress conditions can alter epigenetic regulation, and this can lead to a massive transposition of TEs. Hence, TEs contribute to genome and methylome rearrangements that influence the differentiation of even closely related species or subspecies [4]. A very detailed analysis of five very closely related species of *Neurospora* indicated that both TE content and methylation pattern correlated with phylogenetic relationships [5].

However, it is not only transposable elements that contribute to methylome differentiation. Since most plant genomes are also rich in tandem repeats, which are also abundant in the heterochromatin fraction, changes in their copy number during evolution also affect the inter- and intraspecific diversity of methylomes to a great extent. These sequences in particular, evolve at a relatively rapid rate. The study of three Brassicaceae species: *Arabidopsis thaliana*, *Arabidopsis lyrata*, and *Capsella rubella*, showed that methylome differences are mainly associated with centromere sequences amplifications and deletion of repetitive sequences and TEs. The loss of three centromeres in *A*. *thaliana* relative to the other two species had a significant effect on the methylome and on 5-mC distribution [6].

Both variations in the copy numbers of TEs and of repetitive sequences provide for a large degree of genetic variation, accompanied by a high level of epigenome variation. Nevertheless, some research has shown that significant differences in methylomes may also occur, with relatively low genetic diversity. In the case of *Laguncularia racemosa*, in which significant morphological differences were observed between individuals from salt marshes and river banks, a high diversity of methylomes but a low degree of genetic variability, was found [7]. Similarly, a large degree of plasticity in adaptation to changing environmental conditions, which was a consequence of changes in DNA methylation, was observed in *Pinus pinea* [8]. Studies involving over 1000 different *Arabidopsis* accessions showed a high degree of variability in their methylomes [9], which was associated with a response to environmental factors, and this correlated with their geographies and climates of origin [9, 10]. Thus, the hypothesis that epigenetic variation contributes to adaptation is strongly supported [11, 12].

It seems that some spatial and temporal patterns of DNA methylation that are specific for particular cell types/tissues/species exist, but they may be subject to substantial rearrangements that contribute to phenotypic plasticity and differentiation; thus, cytosine methylation is an important evolutionary factor [13] in generating the extensive intra- and interspecific diversity of methylomes [14, 15]. Comparisons of *A*. *thaliana* to *Z*. *mays* show that the best-known methylome, that of *Arabidopsis*, may not be a good representative of all plant species [16, 17]. The global methylation level of a small genome like *Arabidopsis* is about 10%, while in angiosperms possessing larger genomes, genome-wide methylation level is as high as >40% (e.g. *Beta vulgaris*, *Pancratium maritimum*, *Narcissus bulbocodium*), or even somewhere in the region of 70% in *Secale* sp., which has a mean of about 16% among all species tested [13–15, 18, 19]. Genome expansion through evolution is mainly due to enrichment of the genome with non-coding repetitive sequences and transposable elements; i.e., sequences that becomes highly methylated. This means that an increase in the genome size should be accompanied by an increase in the level of methylation. Rye is an excellent example of a genus whose evolution was accompanied by significant enrichment in non-coding repetitive sequences, as they constitute ~90% of the genome [20, 21]. An unexpectedly high level of global cytosine methylation occurs in the *Secale* genus, ranging from 53% in *S*. *strictum* ssp. *strictum*, to 83% in *S*. *cereale* ssp. *segetale* [19]. Based on previous studies, it is estimated that the global level of genomic methylation increases by about 1.07% for each additional 100 Mb [13]. In comparison, *Beta vulgaris* displays a relatively high rate of global methylation (43%) [13], and it has a genome size of slightly over 700 Mb. Such a high level of methylation in *Secale*, with a cytosine methylation rate of over 80%, seems normal when considering that the rye genome size is approximately 8 Gb [22]. On the other hand, the level of global methylation in the beet genome is very similar to that of the maize genome, which is significantly larger (about 2 Gb) [13]. Although a link between the level of methylation and the size of the genome has been made for many plant species [15, 18], it is likely that the proportional increase in cytosine methylation is slightly vanishing above a certain genome size threshold.

There is no simple relationship between methylation level and genome size or heterochromatin content among *Secale* species/subspecies [19]. Heterochromatin in rye is most abundant in the subtelomeric regions, where, alongside with the telomeres, it is visible as telomeric heterochromatin (t-heterochromatin). It should be expected that the pattern of methylated DNA distribution corresponds to the location of heterochromatin [23–26]. However, there are very limited data on the distribution of 5-mC in the mitotic or meiotic chromosomes of rye [27–29]. Additionally, a detailed study on the distribution pattern of 5-methylcytosine (5-mC) in rye chromosomes has not been made. Using an immunofluorescence (IF) technique, we compared the location of 5-methylcytosine-enriched regions in each chromosome of eight *Secale* taxa. These species/subspecies differ in terms of genome size, global methylation level, and heterochromatin content. This allowed regularities to be established with regard to the overall DNA methylation pattern in rye metaphase chromosomes.

## Materials and methods

### Plant material

The analyses carried out in this study included the following species/subspecies of rye: *Secale cereale* ssp. *cereale*, *Secale cereale* ssp. *afghanicum*, *Secale cereale* ssp. *segetale*, *Secale strictum* ssp. *strictum*, *Secale strictum* ssp. *africanum*, *Secale strictum* ssp. *kuprijanowii*, *Secale sylvestre*, and *Secale vavilovii*. Caryopses of all taxa were obtained from the Botanical Garden of the Polish Academy of Science (Warsaw, Poland).

### Slide preparation

Roots from two-day-old seedlings were immersed in 0.01% colchicine solution (Sigma-Aldrich, St. Louis, USA) for 3.5 hr at 16°C. After thorough rinsing with ice-cold distilled water (3×5 min.), the roots were fixed in Carnoy’s solution (absolute ethanol:glacial acetic acid 3:1 v/v) for 24 h at 4°C. In order to obtain good quality preparations, a two-step maceration was made. First, roots were incubated in 1 N HCl solution for 1 h at room temperature, and after washing with distilled water, an enzyme mixture was used. The mixture contained 5% (w/v) pectinase (Fluka, Buchs, Switzerland), 6% (w/v) hemicellulase (Sigma-Aldrich), and 5% (w/v) cellulase (Sigma-Aldrich) in 0.01 M citric acid–sodium citrate buffer (pH 4.8). The maceration process was performed for 3 h at 37°C, until root tips detached from the roots. The root tips were carefully washed with 0.01 M citric acid–sodium citrate buffer (pH 4.8) and then immersed in 45% acetic acid. Root tips were singly squashed on a microscope slide, in a drop of 45% acetic acid, and covered with a cover glass. Preparations were heated for 15 min. at 47°C. The cover slips were removed after freezing over dry ice, and the slides were air-dried overnight.

### Immunofluorescence

100 μL of formamide solution (70% formamide; 2×SSC, pH 7.0) was applied onto each slide preparation, covered with a cover glass, and placed on a heating block for 2 min. at 70°C. The cover slips were removed, the preparations were dehydrated in an ice-cold ethanol series (70%, 96% and 99.8%), and air-dried. 50 μL of blocking solution (5% BSA; 1×PBS, pH 7.4; 0.05% Tween 20) was applied to each preparation. Slides were incubated for 2 h at room temperature in a humid chamber. Cover slips were removed, and 50 μL of primary antibody solution (2 μg/μL in 1% BSA; 1×PBS, pH 7.4) was added. The main primary antibody used was 5-methylcytosine recombinant rabbit monoclonal antibody (Catalog **#** MA5-24694; Invitrogen, Waltham, USA); additionally, in *S*. *segetale*, anti-5-methylcytosine mouse monoclonal antibody (Catalog **#** A3002; Zymo Research, Irvine, USA) was tested. Incubation with the primary antibody lasted for 1 h at 37°C in a humid chamber. Washes were done in 1×PBS at room temperature, for 3 × 5 min. Thereafter, 50 μL secondary antibody solution (4 μg/μL in 1% BSA; 1×PBS, pH 7.4) was applied to each slide. The following secondary antibodies were used: Goat anti-rabbit IgG (H+L) cross-adsorbed secondary antibody conjugated with Alexa Fluor 488 (Invitrogen), and goat anti-mouse igG1 cross-adsorbed secondary antibody conjugated with Alexa Fluor 647 (Invitrogen). The slides were incubated for 1 h at 37°C in the dark, in a humid chamber. Washes were done in 1×PBS at room temperature, for 3 × 5 min. A small drop of Fluoroshield^TM^ with DAPI (Sigma-Aldrich) was used as a mounting solution, with counterstain.

### Image acquisition and analysis

Preparations were analyzed under an Axio Imager Z2 epifluorescence microscope (Carl Zeiss, Oberkochen, Germany). The images were captured and analyzed using the GenASIs software (Applied Spectral Imaging). From each taxon, 30 preparations were analyzed. NIS-Elements AR 3.1 (Nikon, New York, USA) software was used to measure the total length of the chromosomes, the length of chromosome arms, the run length of telomeric heterochromatin, and the run length coverage of the 5-mC fluorescent signal on chromosomes. Measurements were made in five metaphase plates per taxon. Based on measurements, the extent of the t-heterochromatin regions and the extent of the distal regions deprived of 5-mC in the chromosomes were calculated. For statistical analysis. Fisher’s exact (F) test was used. The Pearson correlation coefficient was calculated using Microsoft Excel.

## Results

The rye genome is characterized by the presence of seven pairs of submetacentric chromosomes that can be easily distinguished based on their differences in length, as well as by the presence and sizes of constitutive heterochromatin bands. DAPI bands indicate the locations of AT-rich heterochromatin, which in rye, are located mainly in the subtelomeric domains and the C bands fully overlap with the DAPI bands. The *Secale* taxa analyzed in this study differed in the presence and size of individual bands which reflected differences in the amount of constitutive heterochromatin in the chromosomes. Thus, it was possible to determine the t-heterochromatin content and DNA methylation patterns in individual chromosomes in each taxon.

The results of this study confirm that 5-methylcytosine comprise a significant fraction of the rye genome. Abundant, dispersed 5-mC signals along all of the chromosomes arms were observed in each *Secale* species. Regions with a slightly higher or lower density of 5-mC could be distinguished. However, most characteristically, the telomeres and subtelomeric constitutive heterochromatin lacked 5-mC. Moreover, centromeric sequences also appeared unmethylated. Detailed characteristics of the 5-mC distribution patterns in all taxa studied are described below.

**Fig 1:**
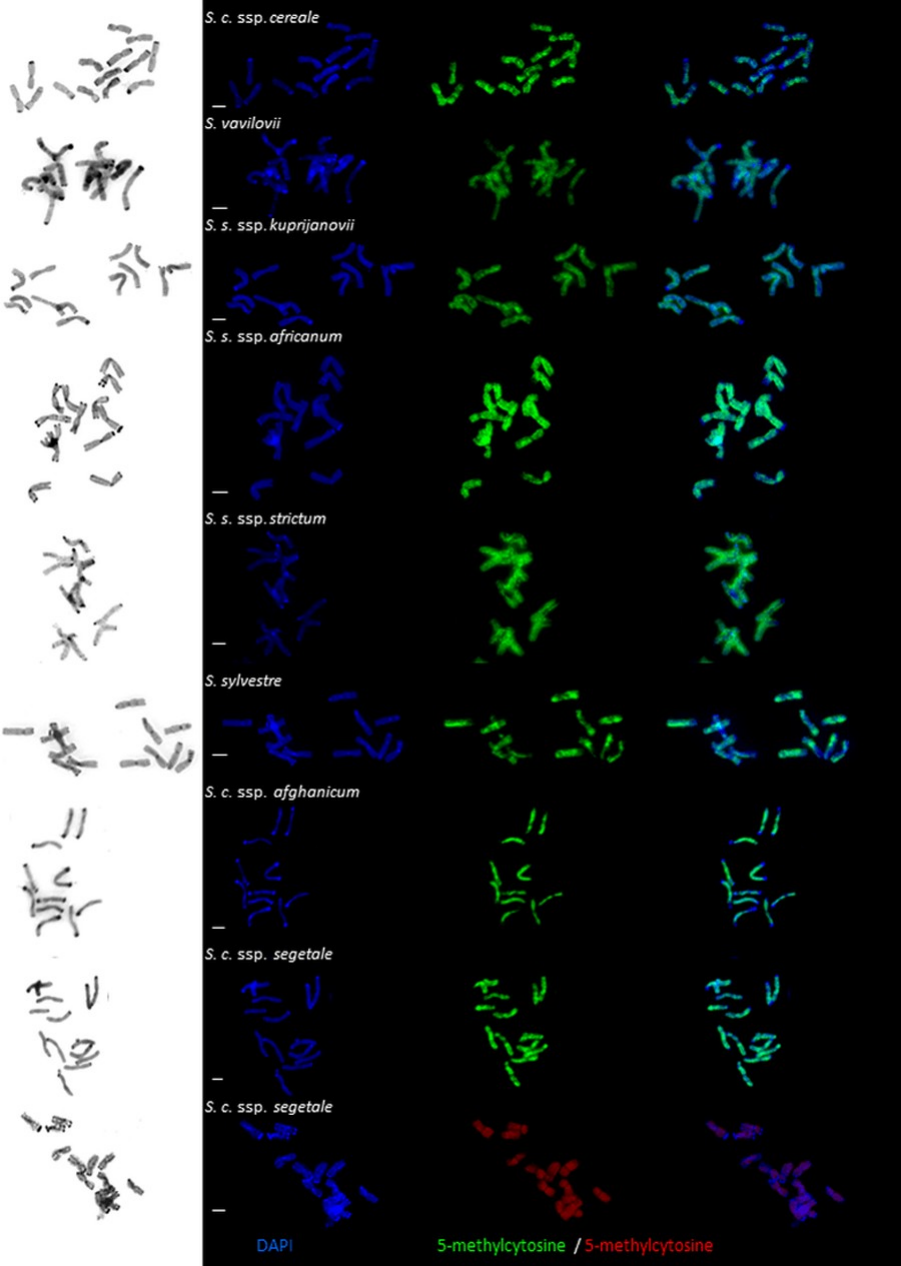
Representative 5-methylcytosine distribution patterns in metaphase chromosomes of *Secale*. Images for inverted DAPI, DAPI, and 5-methylcytosine are shown separately and merged. *Secale cereale* ssp. *cereale*, *Secale vavilovii*, *Secale strictum* ssp. *kuprijanovii*, *Secale strictum* ssp. *africanum*, *Secale strictum* ssp. *strictum*, *Secale strictum*, *Secale cereale* ssp. *afghanicum*, *Secale cereale* ssp. *segetale*. Scale bars represent 5 μm.

5-mC signals occurred on nearly the entire length, in all chromosomes arms, and the observed differentiations between taxa were mainly due to the presence of subtelomeric heterochromatin (Fig 1). Therefore, the 5-mC signal coverage of a given chromosome differed between the taxa tested (Table 1). The 5-mC signal was detectable nearly throughout the length of the chromosomes in *S*. *sylvestre*, a species with the lowest content of subtelomeric heterochromatin (93.95% of total length of the chromosomes). Hence, in species with the highest heterochromatin contents, the 5-mC signal occupied a smaller part of the chromosome length. The lowest rates of 5-mC signal coverage in chromosomes were found in *S*. *cereale* ssp. *segetale* (88.76%), and *S*. *vavilovii* (88.83%). Significant differences in the 5-mC signal coverage length in chromosomes were found between *Secale* taxa (Table 2).

**Table 1:** 5-mC signal coverage of chromosomes length in rye species / subspecies.

Taxon	5-mC signal coverage of chromosomes (% of the chromosome length)							
	Total	1R	2R	3R	4R	5R	6R	7R
***S*. *vavilovii***	88.8	86.1	88.0	87.4	90.3	93.2	89.9	87.5
***S*. *c*. ssp. *cereale***	90.4	90.7	88.5	87.4	94.4	94.0	94.5	87.1
***S*. *c*. ssp. *segetale***	88.8	86.5	86.9	86.7	92.6	92.3	91.5	86.6
***S*. *c*. ssp. *afghanicum***	90.6	91.0	88.8	88.8	93.9	92.2	93.6	87.2
***S*. *sylvestre***	93.9	92.5	93.0	94.8	94.6	94.0	95.2	93.2
***S*. *s*. ssp. *strictum***	92.6	89.1	88.9	97.2	90.1	98.4	96.9	89.5
***S*. *s*. ssp. *africanum***	92.1	92.0	92.7	93.1	95.4	94.1	95.1	84.9
***S*. *s*. ssp. *kuprijanovii***	92.6	93.3	87.5	87.5	97.3	92.2	94.3	91.2

**Table 2:** *P*-values from F test for the differences in 5-mC signal coverage between the chromosomes of rye taxa.

	*S*. *vavilovii*	*S*. *c*. ssp. *segetale*	*S*. *c*. ssp. *afganicum*	*S*. *c*. ssp. *cereale*	*S*. *sylvestre*	*S*. *s*. ssp. *strictum*	*S*. *s*. ssp. *africanum*	*S*. *st*. ssp. *kuprijanovii*
***S*. *vavilovii***		0.81928	0.021006*	0.008479*	4.54E-12*	2.02E-05*	9.32E-06*	2.6897E-09*
***S*. *c*. ssp. *segetale***	0.81928		0.011664*	0.005084*	2.28E-10*	7.45E-06*	8.4E-06*	8.0102E-10*
***S*. *c*. ssp. *afganicum***	0.021006*	0.011664*		0.304304	4.36E-06*	0.016212*	0.01585*	0.00081248*
***S*. *c*. ssp. *cereale***	0.008479*	0.005084*	0.304304		8.25E-05*	0.103327	0.085667	0.01538838*
***S*. *sylvestre***	4.54E-12*	2.28E-10*	4.36E-06*	8.25E-05*		0.212821	0.074281	0.04880178*
***S*. *s*. ssp. *strictum***	2.02E-05*	7.45E-06*	0.016212*	0.103327	0.212821		0.914389	0.97939795
***S*. *s*. ssp. *africanum***	9.32E-06*	8.4E-06*	0.01585*	0.085667	0.074281	0.914389		0.66197291
***S*. *s*. ssp. *kuprijanovii***	2.69E-09*	8.01E-10*	0.000812*	0.015388*	0.048802	0.979398	0.661973	

* statistically significant differences (*P* < 0,05).

The lowest levels of 5-mC signal coverage in chromosomes were observed in 2R and 7R, which in most rye taxa, are characterized by the occurrence of large blocks of telomeric heterochromatin. On the other hand, 4R, 5R, and 6R, which do not have large t-heterochromatin bands on the long arms, were mostly covered with a 5-mC signal (Table 1).

**Fig 2:**
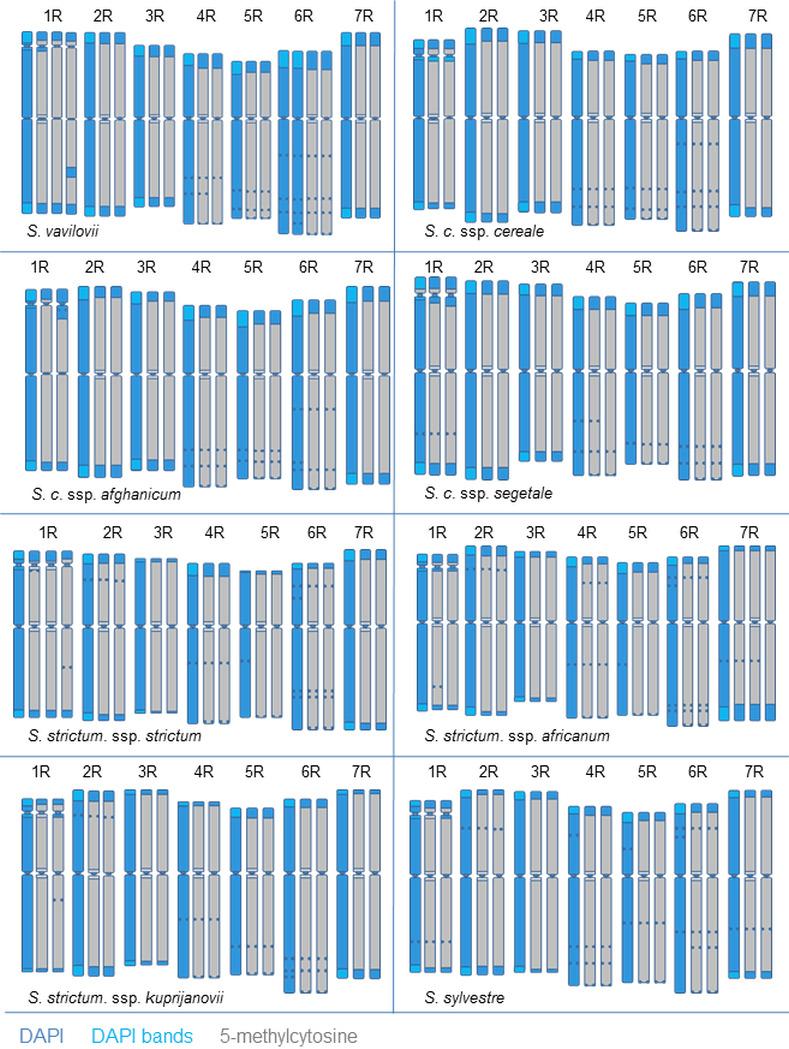
Ideogram showing the distribution of DAPI bands, and the 5-methylcytosine distribution patterns in the metaphase chromosomes of *Secale* species/subspecies. The polymorphism of 5-mC distribution in each chromosome is shown. Ideograms of haploid sets of chromosomes.

Detectable differences in the coverage, distribution, and intensity of the immunofluorescence signal, corresponding to the localization of 5-methylcytosine between the same chromosomes belonging to different taxa, were mainly associated with DAPI band polymorphisms (Fig 2). Furthermore, differences in the 5-mC distribution were also observed among plants in each taxon. These differences were mainly related to the intensity of signals (especially in the pericentromeric region) and the sizes of the distal chromosome regions that lacked 5-mC.

On the basis of the DAPI banding patterns, it was found that there was no cytosine methylation in the telomeric regions, nor in any short chromosome arms encompassing at least part of the subtelomeric region. Chromosome fragments devoid of 5-mC clearly co-localized with subtelomeric heterochromatin DAPI bands (Figs 1 and 2). This was clearly shown by the correlation coefficient, which took into account the range of occurrence of t-heterochromatin and the absence of a 5-mC signal at the chromosome ends. The value of the coefficient was high in most taxa (*S*. *cereale* ssp. *segetale*, *S*. *vavilovii*, *S*. *cereale* ssp. *cereale*, *S*. *cereale* ssp. *afghanicum*), or very high (*S*. *strictum* ssp. *strictum*, *S*. *strictum* ssp. *africanum*, *S*. *strictum* ssp. *kuprijanovii*) (Table 3). A low correlation at 0.231 (a clear relationship) was found in *S*. *sylvestre*, which resulted from a large discrepancy in the obtained measurements among different metaphase spreads, especially the regions deprived of 5-mC in the distal parts of the chromosome arms. Such divergence between chromosomes was not observed in the other analyzed rye taxa.

**Table 3:** Correlation between the extent of t-heterochromatin regions and the extent of distal regions deprived of 5-mC in chromosomes.

Taxon	Chromosome	telomeric heterochromatin	distal region deprived of 5-mC	Correlation coefficient
		(% of the chromosome length)	(% of the chromosome length)	
***S*. *vavilovii***	1R	12.69	13.81	0.744**
	2R	11.35	11.95	
	3R	13.42	12.89	
	4R	6.60	9.61	
	5R	8.07	6.85	
	6R	8.55	10.18	
	7R	12.63	12.56	
***S*. *cereale* ssp. *cereale***	1R	10.64	9.28	0.790**
	2R	14.33	11.47	
	3R	12.43	12.63	
	4R	4.70	5.69	
	5R	3.97	6.07	
	6R	4.40	5.60	
	7R	14.17	12.93	
***S*. *c*. ssp. *segetale***	1R	13.67	13.61	0.742**
	2R	12.27	13.10	
	3R	12.99	13.37	
	4R	6.50	7.61	
	5R	8.05	7.80	
	6R	9.18	8.56	
	7R	13.47	13.84	
***S*. *c*. ssp. *afghanicum***	1R	10.84	9.24	0.794**
	2R	12.60	11.28	
	3R	13.42	11.96	
	4R	7.55	6.21	
	5R	8.58	7.89	
	6R	7.55	6.37	
	7R	14.03	12.35	
***S*. *sylvestre***	1R	7.38	7.62	0.231*
	2R	8.36	6.99	
	3R	7.45	5.16	
	4R	4.21	5.45	
	5R	6.40	6.04	
	6R	5.37	4.85	
	7R	6.98	6.87	
***S*. *s*. ssp. *strictum***	1R	10.62	11.19	0.922***
	2R	13.14	11.38	
	3R	3.22	2.95	
	4R	8.71	9.70	
	5R	0.98	1.80	
	6R	3.73	3.51	
	7R	11.03	10.20	
***S*. *s*. ssp. *africanum***	1R	9.76	8.04	0.929***
	2R	10.10	7.18	
	3R	7.24	6.89	
	4R	6.47	4.63	
	5R	6.35	5.93	
	6R	6.31	4.96	
	7R	14.01	14.54	
***S*. *s*. ssp. *kuprijanovii***	1R	7.09	6.90	0.956***
	2R	13.83	12.55	
	3R	7.42	7.04	
	4R	2.77	2.60	
	5R	7.44	7.83	
	6R	5.27	5.65	
	7R	9.18	8.91	

* 0.2–0.4—weak correlation,

**0.6–0.8—strong correlation,

***0.9–1.0—very strong correlation.

**Fig 3:**
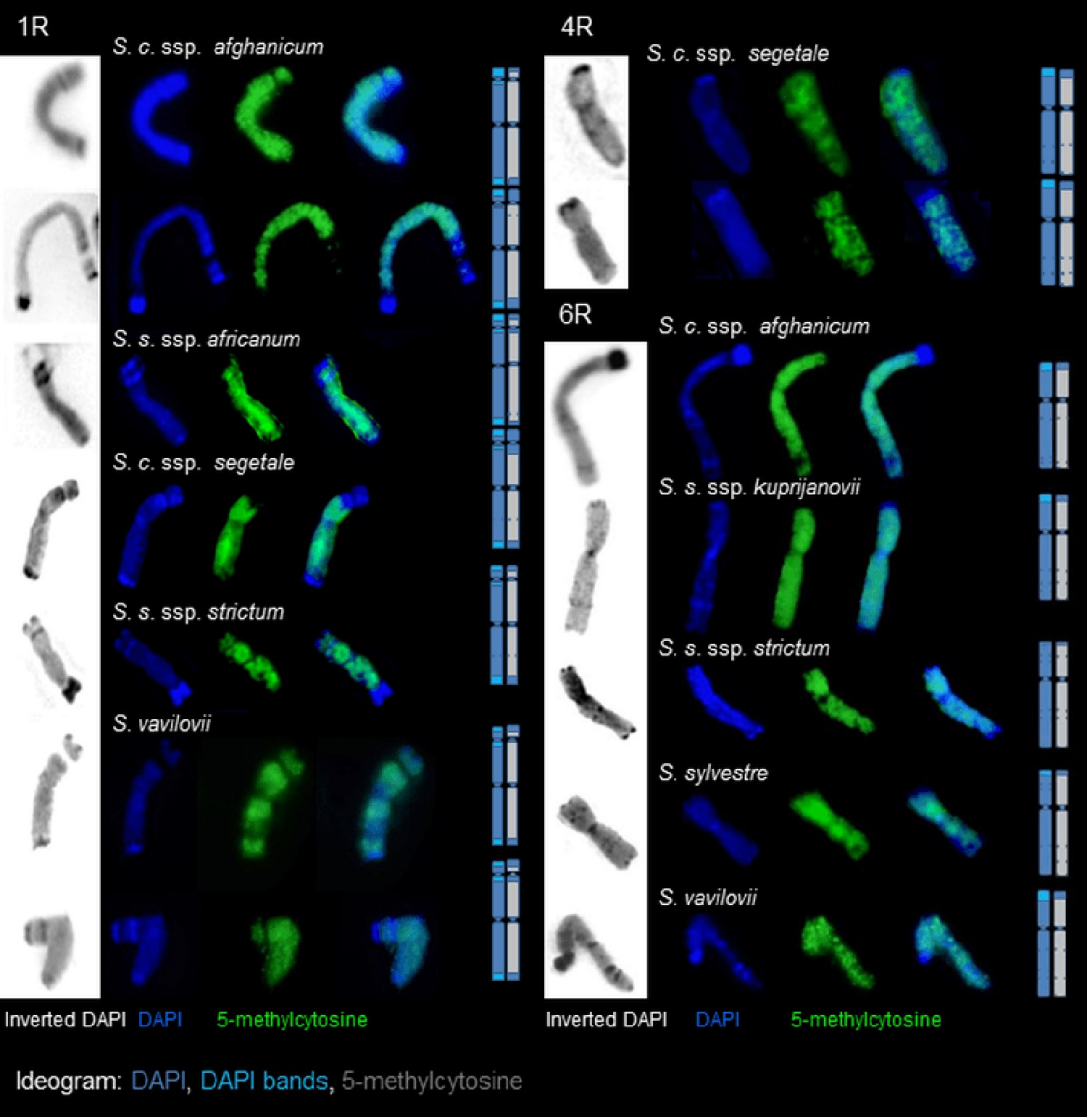
High levels of polymorphism in the distribution of 5-mC in chromosome 1R, 6R and 4R. Images for inverted DAPI, DAPI and 5-methylcytosine are shown separately, as well as merged. Chromosomes 1R: *Secale cereale* ssp. *afghanicum*, *Secale strictum* ssp. *africanum*, *Secale cereale* ssp. *segetale*, *Secale strictum* ssp. *strictum*, *Secale vavilovii*; Chromosomes 4R: *Secale cereale* ssp. *segetale*; Chromosomes 6R: *Secale cereale* ssp. *afghanicum*, *Secale strictum* ssp. *kuprijanovii*, *Secale strictum* ssp. *strictum*, *Secale sylvestre*, *Secale vavilovii*.

Rye chromosomes 4R, 5R, and 6R are characterized by the presence of intercalary DAPI bands in the long arms. In some of these bands, the presence of 5-mC was revealed, while in others, it was not present (e.g., 4RL in *S*. *cereale* ssp. *segetale* or 6RL in *S*. *cereale* ssp. *afghanicum*, *S*. *vavilovii*, *S*. *sylvestre*, and in all subspecies of *S*. *strictum*) (Figs 2 and 3). Occurrences of polymorphism were observed, not only between different taxa, but also between plants in a single taxon. An example of this polymorphism was seen in 7RL of *S*. *strictum* ssp. *africanum*, or 6RL of *S*. *cereale* ssp. *afghanicum* and *S*. *strictum* ssp. *strictum* (Figs 2 and 3).

High levels of polymorphism in the distribution of 5-mC were found in chromosome 1R. This variation was observed both between taxa, and within each taxon. This was especially true for the rate of cytosine methylation in the secondary constriction region and its surroundings (Figs 2 and 3). In addition, the occurrence of 5-mC on 1RS at NOR was accompanied by different intensities of the immunofluorescent signal (Fig 1).

The results indicate a lack of DNA methylation in the centromere region for all rye taxa tested (Figs 1 and 2). It is probable that only the core centromere is unmethylated. Again, due to the imperfection of the technique (high-intensity signals from the pericentromeric areas) the degree of methylation was not visible for each chromosome/metaphase plate. In this case, the Cy5 fluorochrome was much more useful than FITC, which showed a weaker fluorescence signal using the technique (Fig 1), hindering image acquisition. Ambiguous results were obtained in the pericentromeric regions of rye chromosomes. Polymorphisms were observed in each taxon, and this affected each pair of chromosomes. The signal intensity of 5-mC in the pericentromeric region was either comparable to the intensity of the signal in the chromosome arms, or slightly lower (Figs 1 and 2).

As mentioned above, *in situ* techniques may show reduced accuracy. Therefore, we tested another pair of anti-5-methylcytosine primary and secondary antibodies in one taxon. A comparison of 5-mC immunolocalization with different antibodies in *S*. *cereale* ssp. *segetale* did not show any differences. The results with regard to the localization of the 5-mC signal in chromosomes were actually identical (Fig 1). The only difference in the results was the lack of methylation for the DAPI band on chromosome 4RL, which is closer to the centromere.

## Discussion

Due to the extremely high content of non-coding sequences, and the vast accumulation of transposable elements [21] the rye genome should present a very high level of 5-mC, which was proven in a previous study [19]. In order to further analyze the methylation of cytosine in rye, we performed a study of 5-mC distribution in *Secale* mitotic metaphase chromosomes. Earlier, the results of the distribution of methylated cytosine in triticale (indirectly in rye) were presented [29]. However, that study did not clearly show the methylation status of telomeric, centromeric, or NOR regions, with 5-mC being distributed uniformly along the chromosomal arms, and some areas showing a higher density of methylation. Similar results were observed in a study of B chromosomes in rye [27]. This distribution pattern was uniform and punctuated along both A and B chromosomes. Regions rich or lacking 5-mC were not indicated.

We could clearly define a general pattern of 5-methylcytosine distribution in rye, but we observed some inter- and intraspecific differences. The fact that rye species/subspecies have a significant variation in chromosomal cytosine methylation at the global level [19], and not so much variation in the distribution pattern of 5-mC, suggests considerable differences in the number of silenced repetitive sequences, mainly retrotransposons. The 5-mC distribution pattern mostly coincides with the pattern of retrotransposon distribution in rye chromosomes, indicating a high degree of methylation in these sequences. *Ty1-copia* retrotransposons in *S*. *cereale* are distributed throughout the length of the chromosomes, with regions of lower or higher concentration in all chromosomes, as well as in centromeres, except for large subtelomeric heterochromatic blocks. The pattern of *Ty3-gypsy* retrotransposon distribution is similar [30]. Unfortunately, there are no comparisons in the literature with regard to the genetic content of mobile elements in different rye taxa. Such data could help with our understanding of these differences, especially in terms of their global methylation levels.

In contrast to the chromosome arms, the subtelomeric heterochromatin regions were unmethylated. Many eukaryotic chromosomes, including rye, have subtelomere regions, which generally contain specific types of sequences that are distinct from the telomere repeat sequences. *Secale* evolution was accompanied by an enrichment in heterochromatin, and individual species differ in heterochromatin quantity. This is particularly noticeable in the amount and distribution of subtelomeric heterochromatin. Subtelomeric heterochromatin in rye consists mostly of specific families of repetitive sequences [31], with the most abundant being pSc119.2, pSc200, and pSc250 [32]. Of these, the pSc119.2 sequences are located most proximally to the subtelomeric region, while pSc200 and pSc250 sequences are distributed more distally [32, 33]. Specific domains containing pSc200 and pSc250 can be distinguished, with some parts overlapping [32, 34, 35]. Sequences belonging to the pSc119.2 and pSc200 families are present in the subtelomeric regions of all chromosomes, whereas the pSc250 sequences are absent from some subtelomeric regions [36]. Apparently, pSc119.2, pSc200 and pSc250 remain unmethylated, at least for those found in the subtelomeric region. The pSc119.2 sequences may be present in the intercalary regions of the chromosomes [32, 34], and their methylation status can be different from the copies located in the subtelomeric region. It should be noted that all of these sequences contain a large number of cytosines (the most numerous being CG motifs, but with CHG and CHH also present); thus, they can potentially be methylated. Considering the number of repeats of these sequences in most of the subtelomeres, their methylation should be considered at the cytogenetic level. However, we easily observed with 100% repeatability the absence of the 5-mC signal in this region of chromosomes, in each taxon studied. Therefore, the lack of methylation in this area is not due to the imperfection of the IF method. This finding also gives a clear explanation for why the global level of methylation in different rye species/subspecies is not correlated with the amount of t-heterochromatin [19]. This indicates that a very high global level of DNA methylation is due to its high density in other areas of the chromosomes.

It is possible that the absence of 5-methylcytosine in the subtelomeric region is due to its potentially important role in ensuring chromosome homeostasis and gene expression within these heterochromatic regions; i.e., providing a buffer zone against heterochromatin spread into neighboring euchromatin regions [37]. In *Schizosaccharomyces pombe*, a part of the subtelomere region forms a “knob”, which is a highly condensed chromatin structure. However, this region does not represent typical heterochromatin [38]. In this region, the levels of histone modifications are much lower compared to the rest of the subtelomeric heterochromatin and the adjacent euchromatin [39]. More frequently it is being shown that heterochromatin is heterogeneous, characterized by plasticity, and that its epigenetic regulators depend on the genomic context in which it is present. This may also be related to the fact that the sequences of some constitutive heterochromatin regions are transcribed [40]. The subtelomeric chromatin fraction, like the telomeric or centromere fractions, may be a type of intermediate chromatin, with unique epigenetic markers. Perhaps these are telomere border regions, which, by the amplification of a certain type of sequence, are clearly distinguishable in most chromosome arms in rye, with their size allowing for the level of their methylation to be assessed. In the arms of chromosomes lacking large blocks of subtelomeric chromatin, it is possible that much smaller copy numbers for these sequences are capable of fulfilling the same role, but this region is indistinguishable from the telomeres.

It has been shown that heterochromatin is not stable, and its dynamic nature has been indicated through various studies [41]. Moreover, other studies have undermined the prevailing belief that the heterochromatin fraction is always highly methylated. For example, in aphids (*Acyrthosiphon pisum*) cytosine residues in constitutive heterochromatin are not methylated, whereas the euchromatin is methylated [42]. In *Vicia faba*, the chromosomal distribution of 5-mC was mostly unrelated to C-band localization [43]. Contrary to the prevailing view that associates heterochromatin with high levels of cytosine methylation, that late replicating regions have been observed as being less heavily methylated than the early replicating regions in the human genome [51]. Based on these results, it has been concluded that the relationship between cytosine methylation in DNA and heterochromatin structures is not as simple as was first assumed.

For many years, centromeres and telomeres were regarded as typically heterochromatic regions. It is currently being suggested that plant telomeres are not conventional heterochromatin structures, as they may simultaneously possess the characteristics of both euchromatin and heterochromatin [44, 45]. Some studies even indicate that telomeres can exhibit mainly euchromatic traits, while subtelomeres exhibit heterochromatic traits [46]. Methylation studies of telomeric sequences, as with studies of the post-translational modifications of histones, have yielded ambiguous results. In *Arabidopsis thaliana*, one study showed the presence of methylation in telomeric sequences [47], whereas another study indicated the lack of methylation in this region [48]. In turn, DNA methylation of the telomeric and subtelomeric regions in three cotton species (*G*. *hirsutum*, *G*. *herbaceum* and *G*. *arboreum)* showed interspecific variation [49].

As with the telomere region, the results of research on the epigenetic status of the centromeric region are not consistent. An analysis of the methylation status of the human neocentromere showed general hypermethylation of sequences in this region [50]. Centromeric sequences in plants were also considered to be highly methylated [51]. However, some reports have clearly contradicted this notion. In *Arabidopsis*, centromeric chromatin sequences have been shown to be hypomethylated, in contrast to the same repetitive sequences when located in the flanking pericentromeric heterochromatin [24]. Similarly, hypomethylation of the centromeric DNA sequence was found in maize [24]. In this study, we have shown that in rye, the centromeric DNA is not methylated, while the adjacent pericentromere regions exhibit varying levels of methylation. Methylation patterns in the centromere region also exemplify heterochromatin heterogeneity. Both the centromere and pericentromere are largely composed of retrotransposons that mainly belong to the *Ty3-gypsy* family [52]. While the centromeres remain unmethylated, the pericentromeres show hypermethylation. Also, in this case, the degree of methylation can be potentially explained by the different functionalities of these areas.

A polymorphism in the distribution and intensity of 5-mC signals was observed between rye taxa, and even between individual samples. Regions in which 5-mC always occurred could be distinguished, as well as regions where the presence and pattern of 5-mC was not regular. 5-methylcytosine immunostaining often presents ambiguous results, and there can be differences in the distribution and intensity of signals between cells, or even between homologous chromosomes [29, 43, 53, 54]. However, it appears that a much larger number of subtle factors are involved in the observed diversity, as even homologous chromosomes may show variable rates of transcriptional activity in corresponding regions [55].

Epigenomic variation is shaped by genomic structural variation, and genomic or epigenomic changes have reciprocal effects [9]. Importantly, however, an understanding of epigenome evolution is a prerequisite for conducting inter- and intraspecific studies in comparative epigenomics. The results of this study clearly indicate the need for further analyses using other species, especially those with larger genomes.

## Conclusions

The studied species and subspecies of *Secale* have a very similar pattern of distribution of 5-methylcytosine in chromosomes, which, agree with genetic studies of the *Secale* genus, in demonstrating the high taxonomic similarity between its different species and subspecies. A lack of cytosine methylation in the telomeric and centromeric regions confirms that, despite the high level of condensation, this is not typical heterochromatin, but a type of intermediate chromatin, with a unique set of epigenetic tags. Moreover, there was a complete absence of 5-mC in the subtelomeric region, which was considered to correspond to constitutive heterochromatin. It can be hypothesized that these regions have some specific function, for which the maintenance of an appropriate set of epigenetic markers must be ensured. Thus, subtelomeres do not represent typical constitutive heterochromatin, and their genetic and epigenetic characteristics may be species or generic specific. The presence of 5-mC signals of varying intensity along the arms of the chromosomes indicates the presence of hypermethylation at the retrotransposon sequences, which are abundant within these areas. A certain level of polymorphism, especially in the case of chromosome 1R, was observed, not only between different taxa, but also between plants in one taxon, which may indicate differences in the activity of these chromosomal regions.
